# Supplementary material for: Glioblastoma remodelling of human neural circuits decreases survival
Source: Nature. 2023 May 3;617(7961):599–607. doi: 10.1038/s41586-023-06036-1 (PMC10191851; doi:10.1038/s41586-023-06036-1)
Supplement: Supplementary file 1 — Supplementary Tables 1–5. [file 41586_2023_6036_MOESM1_ESM.docx]

**Supplementary Table 1.** List of patient samples used for each experiment.

| **Patient #** | Human ECoG | RNA Transcriptomics | Human Tissue  IHC+ IF | 1° Patient Cell Culture | Protein Analysis  (Proteomics+ ELISA) | PDX | Language Tasks | Human Survival |
| --- | --- | --- | --- | --- | --- | --- | --- | --- |
| SF#1 |  |  |  |  |  |  |  | ✓ |
| SF#2 |  |  |  |  |  |  |  | ✓ |
| SF#3 |  |  |  |  |  |  |  |  |
| SF#4 |  |  |  |  | ✓ |  | ✓ | ✓ |
| SF#5 |  |  |  |  |  |  | ✓ |  |
| SF#6 |  |  |  |  |  |  | ✓ | ✓ |
| SF#7 |  |  |  |  |  |  | ✓ | ✓ |
| SF#8 | ✓ |  |  |  |  |  |  |  |
| SF#9 |  |  |  |  | ✓ |  | ✓ | ✓ |
| SF#10 |  |  |  |  |  |  |  | ✓ |
| SF#11 | ✓ |  |  |  |  |  |  |  |
| SF#12 |  |  |  |  | ✓ |  | ✓ | ✓ |
| SF#13 |  |  |  |  |  |  |  | ✓ |
| SF#14 |  |  |  |  |  |  |  | ✓ |
| SF#15 |  |  |  |  |  |  |  |  |
| SF#16 | ✓ |  |  |  |  |  |  |  |
| SF#17 |  |  |  |  |  |  |  | ✓ |
| SF#18 |  | *✓ | *✓ |  | ✓ |  |  |  |
| SF#19 |  |  |  |  | ✓ |  |  | ✓ |
| SF#20 |  |  |  |  |  |  | ✓ |  |
| SF#21 |  |  |  |  | ✓ |  |  |  |
| SF#22 |  |  |  |  | ✓ |  | ✓ | ✓ |
| SF#23 |  |  |  |  | ✓ |  | ✓ | ✓ |
| SF#24 |  |  |  |  |  |  |  | ✓ |
| SF#25 |  |  |  |  |  |  |  | ✓ |
| SF#26 | ✓ |  |  |  |  |  |  |  |
| SF#27 |  |  |  |  | ✓ |  |  | ✓ |
| SF#28 | ✓ |  |  |  |  |  |  |  |
| SF#29 | ✓ | *✓ | *✓ | *✓ | *✓ |  | ✓ | ✓ |
| SF#30 |  |  |  |  |  |  | ✓ | ✓ |
| SF#31 |  |  |  | *✓ | *✓ |  |  | ✓ |
| SF#32 |  | ✓ |  | ✓ |  |  |  | ✓ |
| SF#33 |  |  | ✓ | ✓ | ✓ | ✓ |  | ✓ |
| SF#34 |  | ✓ | ✓ | ✓ | ✓ |  | ✓ | ✓ |
| SF#35 |  | ✓ | ✓ | ✓ |  | ✓ | ✓ | ✓ |
| SF#36 |  |  |  | ✓ |  |  |  | ✓ |
| SF#37 |  |  |  |  | ✓ |  | ✓ | ✓ |
| SF#38 |  |  |  |  |  | ✓ |  | ✓ |
| SF#39 |  |  | ✓ |  |  |  |  | ✓ |
| SF#40 | ✓ |  |  |  |  |  |  |  |
| SF#41 |  |  |  |  |  |  | ✓ |  |
| SF#42 |  |  |  |  |  |  |  | ✓ |
| SF#43 |  |  |  |  | ✓ |  | ✓ | ✓ |
| SF#44 |  |  |  | ✓ |  |  |  | ✓ |
| SF#45 |  |  | ✓ | ✓ | ✓ |  |  |  |
| SF#46 |  |  | *✓ |  |  |  |  |  |
| SF#47 |  |  |  |  | ✓ | ✓ |  | ✓ |
| SF#48 |  |  |  |  |  |  |  | ✓ |
| SF#49 |  |  | ✓ | *✓ |  |  |  |  |
| SF#50 | ✓ |  |  |  |  |  |  |  |
| SF#51 |  |  |  | *✓ |  |  |  |  |
| SF#52 | ✓ |  |  |  |  |  |  |  |
| SF#53 |  |  |  | *✓ |  |  |  |  |
| SF#54 |  | *✓ | *✓ | ✓ |  |  |  |  |
| SF#55 |  |  |  |  | ✓ |  |  |  |
| SF#56 |  |  |  | ✓ |  |  |  |  |
| SF#57 |  | *✓ | *✓ | ✓ |  |  |  |  |
| SF#58 | ✓ |  |  |  |  |  |  |  |
| SF#59 |  | *✓ |  | ✓ |  |  |  |  |
| SF#60 |  |  | *✓ | ✓ |  |  |  |  |
| SF#61 | ✓ |  |  |  |  |  |  |  |
| SF#62 |  |  |  | ✓ |  |  |  |  |
| SF#63 |  |  |  | ✓ |  |  |  |  |
| SF#64 | ✓ |  |  |  |  |  |  |  |
| SF#65 |  |  |  |  |  | ✓ |  |  |
| SF#66 |  |  |  |  |  | *✓ |  |  |
| SF#67 |  |  |  |  |  | ✓ |  |  |
| SF#68 |  |  |  |  |  |  |  | ✓ |
| SF#69 |  |  |  |  |  |  |  | ✓ |
| SF#70 |  |  |  |  |  |  |  | ✓ |
| SF#71 |  |  |  |  |  |  |  | ✓ |
| SF#72 |  |  |  |  |  |  |  | ✓ |
| SF#73 |  |  |  |  |  |  |  | ✓ |
| SF#74 |  |  |  |  |  |  |  | ✓ |
| SF#75 |  |  |  |  |  |  |  | ✓ |
| SF#76 |  |  |  |  |  |  |  | ✓ |
| SF#77 |  |  |  |  |  |  |  | ✓ |
| SF#78 |  |  |  |  |  |  |  | ✓ |
| SF#79 |  |  |  |  |  |  |  | ✓ |
| SF#80 |  |  |  |  |  |  |  | ✓ |
| SF#81 |  |  |  |  |  |  |  | ✓ |
| SF#82 |  |  |  |  |  |  |  | ✓ |
| SF#83 |  |  |  |  |  |  |  | ✓ |
| SF#84 |  |  |  |  |  |  |  | ✓ |
| SF#85 |  |  |  |  |  |  |  | ✓ |
| SF#86 |  |  |  |  |  |  |  | ✓ |
| SF#87 |  |  |  |  |  |  |  | ✓ |
| SF#88 |  |  |  |  |  |  |  | ✓ |
| SF#89 |  |  |  |  |  |  |  | ✓ |
| SF#90 |  |  |  |  |  |  |  | ✓ |
| SF#91 |  |  |  |  |  |  |  | ✓ |
| SF#92 |  |  |  |  |  |  |  | ✓ |
| SF#93 |  |  |  |  |  |  |  | ✓ |
| SF#94 |  |  |  |  |  |  |  | ✓ |
| SF#95 |  |  |  |  |  |  |  | ✓ |
| SF#96 |  |  |  |  |  |  |  | ✓ |
| SF#97 |  |  |  |  |  |  |  | ✓ |
| SF#98 |  |  |  |  |  |  |  | ✓ |
| SF#99 |  |  |  |  |  |  |  | ✓ |
| SF#100 |  |  |  |  |  |  |  | ✓ |
| SF#101 |  |  |  |  |  |  |  | ✓ |
| UM#1 | ✓ |  |  |  |  |  |  |  |
| UM#2 | ✓ |  |  |  |  |  |  |  |
| **Total N** | **14** | **13** | **18** | **24** | **20** | **8** | **16** | **66** |

*✓ Patient-matched samples.

**Supplementary Table 2.** Patient summary-clinical and molecular features

| **Study #** | **Sex** | **Age**  **(yr)** | **Preoperative tumor volume (ml)** | **Residual tumor (ml)** | **EOR (%)** | **MGMT methylation** | **EGFR**  **amplification** | **Tumor Location** | **Tumor type** | **IDH status** |
| --- | --- | --- | --- | --- | --- | --- | --- | --- | --- | --- |
| SF#1 | M | 56.07 | 23.67 | 0.00 | 100.00 | no | nonamp | R frontal | GBM | wt |
| SF#2 | M | 64.93 | 9.20 | 0.00 | 100.00 | yes | amp | L temporal | GBM | wt |
| SF#3 | M | 60.96 | 49.68 | 3.95 | 92.06 | yes | nonamp | L temporal | GBM | wt |
| SF#4 | M | 60.42 | 20.30 | 0.00 | 100.00 | yes | amp | L frontal/insula | GBM | wt |
| SF#5 | M | 76.72 | 8.94 | 0.00 | 100.00 | no | nonamp | L frontal | GBM | wt |
| SF#6 | M | 72 | 23.59 | 4.79 | 79.70 | yes | nonamp | L temporal | GBM | wt |
| SF#7 | F | 64.14 | 78.37 | 0.00 | 100.00 | yes | amp | R frontal | GBM | wt |
| SF#8 | M | 78.12 | 77.49 | 0.00 | 100.00 | yes | amp | L frontal | GBM | wt |
| SF#9 | F | 62.2 | 14.13 | 0.00 | 100.00 | no | amp | L temporal | GBM | wt |
| SF#10 | F | 59.02 | 6.38 | 0.00 | 100.00 | yes | amp | R frontal | GBM | wt |
| SF#11 | M | 57.26 | 46.07 | 1.09 | 97.64 | no | nonamp | L temporal | GBM | wt |
| SF#12 | M | 55.34 | 53.10 | 7.33 | 86.19 | yes | nonamp | L frontal | GBM | wt |
| SF#13 | F | 66.92 | 3.20 | 0.00 | 100.00 | yes | amp | L temporal | GBM | wt |
| SF#14 | M | 29.3 | 36.88 | 0.00 | 100.00 | yes | amp | R insula | GBM | wt |
| SF#15 | M | 51.15 | 29.67 | 2.63 | 91.14 | no | nonamp | L thalamus | GBM | wt |
| SF#16 | M | 48.92 | 31.05 | 0.00 | 100.00 | no | nonamp | L frontal | GBM | wt |
| SF#17 | F | 49.3 | 55.55 | 3.49 | 93.72 | yes | amp | L frontal | GBM | wt |
| SF#18 | M | 60.98 | 67.50 | 0.00 | 100.00 | yes | amp | L frontal | GBM | wt |
| SF#19 | M | 80.1 | 45.41 | 0.75 | 98.35 | yes | nonamp | L parietal | GBM | wt |
| SF#20 | F | 72.89 | 6.12 | 0.00 | 100.00 | yes | nonamp | R frontal | GBM | wt |
| SF#21 | F | 56.34 | 81.00 | 0.00 | 100.00 | yes | amp | L parietal | GBM | wt |
| SF#22 | M | 63.22 | 7.01 | 0.53 | 92.41 | yes | amp | L parietal | GBM | wt |
| SF#23 | F | 60.04 | 15.69 | 0.00 | 100.00 | yes | amp | L parietal | GBM | wt |
| SF#24 | F | 69.62 | 92.46 | 0.00 | 100.00 | no | nonamp | R frontal | GBM | wt |
| SF#25 | M | 60.38 | 101.96 | 0.21 | 99.79 | yes | nonamp | R frontal | GBM | wt |
| SF#26 | F | 52.61 | 7.32 | 0.00 | 100.00 | yes | nonamp | L temporal | GBM | wt |
| SF#27 | M | 52.72 | 41.21 | 3.58 | 91.31 | no | amp | R frontal | GBM | wt |
| SF#28 | F | 59.89 | 9.51 | 0.00 | 100.00 | no | nonamp | L temporal | GBM | wt |
| SF#29 | M | 44.92 | 34.59 | 0.00 | 100.00 | yes | amp | R temporal | GBM | wt |
| SF#30 | F | 65.53 | 15.77 | 0.48 | 96.96 | no | unknown | Parietal | GBM | wt |
| SF#31 | F | 67.89 | 14.14 | 0.00 | 100.00 | yes | nonamp | L multifocal | GBM | wt |
| SF#32 | M | 71.49 | 21.80 | 0.00 | 100.00 | no | amp | L temporal | GBM | wt |
| SF#33 | F | 61.72 | 52.27 | 0.17 | 99.67 | no | amp | R occipital | GBM | wt |
| SF#34 | M | 69.03 | 57.01 | 0.00 | 100.00 | yes | nonamp | L parietal | GBM | wt |
| SF#35 | F | 46.9 | 35.99 | 0.00 | 100.00 | yes | yes | R temporal | GBM | wt |
| SF#36 | M | 51.73 | 37.45 | 2.20 | 94.13 | no | amp | L frontal | GBM | wt |
| SF#37 | M | 69.67 | 59.66 | 0.33 | 99.45 | yes | nonamp | R parieto-occipital | GBM | wt |
| SF#38 | M | 69.06 | 74.40 | 2.49 | 96.65 | yes | nonamp | R parietal | GBM | wt |
| SF#39 | F | 69.83 | 6.85 | 0.17 | 97.51 | yes | amp |  | GBM | wt |
| SF#40 | M | 54.05 | 61.69 | 3.00 | 95.13 | yes | amp | L frontal | GBM | wt |
| SF#41 | F | 57.39 | 9.25 | 0.00 | 100.00 | yes | nonamp | L frontal | GBM | wt |
| SF#42 | M | 60.21 | 101.06 | 0.41 | 99.59 | no | nonamp | L temporal | GBM | wt |
| SF#43 | M | 76.75 | 44.02 | 1.13 | 97.43 | yes | nonamp | R temporal | GBM | wt |
| SF#44 | M | 43.81 | 17.72 | 0.00 | 100.00 | no | amp | R frontal | GBM | wt |

**Supplementary Table 3**. Bulk RNA-seq based detection of the significant differentially expressed genes in high and low connectivity regions with log fold changes greater than 1 and an adjusted *P*-value less than 0.05. *P* value calculated using two-sided Wald test and adjusted for multiple comparisons with the Benjamini-Hochberg method.

| **Gene Name** | **Log2 Fold-change** | ***P-*value** | **Adjusted *P*-value** |
| --- | --- | --- | --- |
| PI3 | 8.99 | 2.78E-13 | 9.71E-10 |
| MARCO | 8.25 | 7.65E-19 | 6.67E-15 |
| TUBB2BP1 | -6.78 | 6.59E-22 | 1.15E-17 |
| SLAMF9 | 6.08 | 3.14E-05 | 7.10E-03 |
| LUCAT1 | 4.97 | 1.59E-06 | 7.10E-04 |
| SLPI | 4.74 | 1.39E-11 | 4.03E-08 |
| PLA2G2A | 4.74 | 3.12E-10 | 6.80E-07 |
| CXCL5 | 4.64 | 2.42E-05 | 5.73E-03 |
| STEAP4 | 4.53 | 1.16E-09 | 2.25E-06 |
| EVA1A | 4.41 | 4.25E-04 | 4.75E-02 |
| CHI3L1 | 4.30 | 1.56E-15 | 6.81E-12 |
| LTF | 4.18 | 1.75E-16 | 1.01E-12 |
| MME | 4.14 | 1.30E-05 | 3.50E-03 |
| GALNT5 | 4.10 | 1.19E-06 | 5.77E-04 |
| SAA2 | 4.10 | 1.48E-04 | 2.17E-02 |
| HP | 4.07 | 1.43E-07 | 1.08E-04 |
| IL6 | 4.06 | 2.31E-09 | 3.52E-06 |
| FGF7 | 3.85 | 3.29E-07 | 2.01E-04 |
| CXCL8 | 3.69 | 2.86E-08 | 2.93E-05 |
| ADAMTS2 | 3.67 | 2.84E-07 | 1.84E-04 |
| CXCL3 | 3.58 | 5.67E-06 | 1.87E-03 |
| NNMT | 3.58 | 2.26E-08 | 2.63E-05 |
| TFPI2 | 3.53 | 2.03E-04 | 2.62E-02 |
| CD300E | 3.49 | 3.71E-05 | 7.82E-03 |
| PRF1 | 3.49 | 3.45E-06 | 1.37E-03 |
| MMP19 | 3.49 | 2.75E-08 | 2.93E-05 |
| LYVE1 | 3.46 | 7.74E-09 | 9.64E-06 |
| MYBPH | 3.39 | 4.82E-06 | 1.68E-03 |
| C7 | 3.38 | 5.19E-09 | 6.96E-06 |
| TREM1 | 3.29 | 1.63E-06 | 7.11E-04 |
| INHBA | 3.26 | 1.38E-07 | 1.08E-04 |
| CHI3L2 | 3.24 | 4.45E-11 | 1.11E-07 |
| AQP9 | 3.24 | 1.03E-06 | 5.26E-04 |
| CA9 | 3.23 | 3.12E-05 | 7.10E-03 |
| ITGBL1 | 3.20 | 1.05E-05 | 3.12E-03 |
| OPALIN | -3.15 | 1.41E-07 | 1.08E-04 |
| ADARB2 | -3.06 | 1.26E-06 | 5.94E-04 |
| ABCA13 | 3.04 | 8.81E-05 | 1.45E-02 |
| IL1A | 2.98 | 3.77E-04 | 4.33E-02 |
| SPOCD1 | 2.98 | 2.30E-09 | 3.52E-06 |
| GPC3 | 2.96 | 5.70E-05 | 1.07E-02 |
| INSRR | 2.95 | 4.26E-05 | 8.53E-03 |
| COL12A1 | 2.95 | 3.86E-06 | 1.43E-03 |
| LIF | 2.93 | 9.92E-07 | 5.26E-04 |
| MEOX2 | -2.93 | 8.00E-07 | 4.50E-04 |
| GPRC5A | 2.91 | 1.78E-05 | 4.44E-03 |
| TSP-1 | 2.89 | 1.02E-06 | 5.26E-04 |
| CAV1 | 2.89 | 2.17E-07 | 1.51E-04 |
| CD163 | 2.89 | 3.34E-07 | 2.01E-04 |
| HK3 | 2.87 | 2.48E-07 | 1.66E-04 |
| SOCS3 | 2.81 | 2.43E-09 | 3.52E-06 |
| BTBD11 | 2.80 | 1.06E-05 | 3.12E-03 |
| ALPK2 | 2.77 | 3.06E-04 | 3.69E-02 |
| CYP1B1 | 2.75 | 4.78E-07 | 2.78E-04 |
| MET | 2.74 | 1.44E-06 | 6.60E-04 |
| FCGR2C | 2.73 | 8.09E-06 | 2.56E-03 |
| VEPH1 | 2.69 | 1.88E-04 | 2.52E-02 |
| FBLN2 | 2.65 | 2.02E-05 | 4.95E-03 |
| LINC01272 | 2.65 | 1.23E-05 | 3.42E-03 |
| GCNT1 | 2.65 | 3.55E-05 | 7.64E-03 |
| HMOX1 | 2.63 | 5.83E-08 | 5.64E-05 |
| IL1R1 | 2.60 | 2.16E-07 | 1.51E-04 |
| F13A1 | 2.58 | 1.74E-05 | 4.43E-03 |
| FAP | 2.56 | 1.65E-05 | 4.29E-03 |
| PLAU | 2.55 | 1.08E-06 | 5.37E-04 |
| RNASE2 | 2.54 | 1.30E-05 | 3.50E-03 |
| COL5A1 | 2.53 | 1.37E-05 | 3.61E-03 |
| STAB1 | 2.51 | 2.54E-06 | 1.05E-03 |
| CCL2 | 2.50 | 8.32E-08 | 7.63E-05 |
| CA12 | 2.50 | 2.40E-05 | 5.73E-03 |
| PTGS2 | 2.48 | 1.01E-04 | 1.63E-02 |
| C1R | 2.48 | 1.19E-07 | 1.04E-04 |
| ACTA2 | 2.48 | 1.05E-05 | 3.12E-03 |
| H19 | 2.47 | 3.62E-06 | 1.40E-03 |
| IER3 | 2.46 | 2.23E-06 | 9.50E-04 |
| HSPA7 | 2.44 | 3.79E-05 | 7.87E-03 |
| CXCL12 | 2.43 | 1.75E-05 | 4.43E-03 |
| TIMP1 | 2.40 | 2.43E-05 | 5.73E-03 |
| IL1R2 | 2.40 | 7.27E-05 | 1.26E-02 |
| SEZ6L | -2.36 | 4.14E-06 | 1.50E-03 |
| IL7R | 2.35 | 1.11E-04 | 1.74E-02 |
| LTBP2 | 2.35 | 4.31E-06 | 1.53E-03 |
| ADAMTSL1 | 2.35 | 6.26E-05 | 1.14E-02 |
| S100A9 | 2.34 | 3.72E-06 | 1.41E-03 |
| AP000892.6 | 2.29 | 1.85E-04 | 2.51E-02 |
| COL6A2 | 2.29 | 3.35E-06 | 1.36E-03 |
| DCN | 2.26 | 3.86E-05 | 7.92E-03 |
| IGF2 | 2.24 | 5.08E-06 | 1.74E-03 |
| ADAMTS12 | 2.22 | 8.11E-05 | 1.37E-02 |
| IL1B | 2.21 | 1.09E-04 | 1.72E-02 |
| S100A4 | 2.17 | 1.76E-04 | 2.46E-02 |
| ANGPTL4 | 2.15 | 8.31E-05 | 1.39E-02 |
| GFPT2 | 2.14 | 1.14E-05 | 3.20E-03 |
| TPBG | 2.13 | 2.16E-04 | 2.76E-02 |
| MYO1G | 2.11 | 1.66E-04 | 2.37E-02 |
| ASPN | 2.10 | 4.24E-04 | 4.75E-02 |
| NHLH1 | 2.08 | 1.70E-04 | 2.41E-02 |
| CAMK2A | -2.06 | 3.48E-05 | 7.59E-03 |
| COL14A1 | 2.03 | 4.58E-05 | 9.07E-03 |
| C5AR1 | 2.02 | 5.89E-05 | 1.09E-02 |
| BIRC3 | 2.02 | 2.03E-04 | 2.62E-02 |
| TGFBI | 2.02 | 5.08E-05 | 9.84E-03 |
| C1S | 2.01 | 7.95E-06 | 2.56E-03 |
| COLEC12 | 2.01 | 6.36E-05 | 1.14E-02 |
| JAK3 | 2.00 | 1.19E-04 | 1.82E-02 |
| FOSL1 | 1.98 | 1.21E-04 | 1.83E-02 |
| OSMR | 1.98 | 1.13E-05 | 3.20E-03 |
| SERPING1 | 1.96 | 9.04E-06 | 2.82E-03 |
| OLFML2B | 1.95 | 7.37E-05 | 1.26E-02 |
| TYMP | 1.93 | 4.88E-05 | 9.56E-03 |
| NAMPT | 1.91 | 6.19E-05 | 1.14E-02 |
| CCL5 | 1.89 | 2.21E-04 | 2.81E-02 |
| ADAM8 | 1.88 | 3.36E-04 | 3.96E-02 |
| DCC | -1.87 | 5.28E-05 | 1.00E-02 |
| MS4A4A | 1.87 | 1.90E-04 | 2.52E-02 |
| ANXA2 | 1.87 | 9.63E-05 | 1.57E-02 |
| CNGA3 | -1.86 | 4.07E-05 | 8.25E-03 |
| CYR61 | 1.85 | 3.18E-05 | 7.10E-03 |
| EMP3 | 1.84 | 6.39E-05 | 1.14E-02 |
| C1RL | 1.84 | 6.53E-05 | 1.15E-02 |
| COL6A1 | 1.84 | 3.07E-05 | 7.10E-03 |
| GBP2 | 1.82 | 3.39E-05 | 7.49E-03 |
| P4HA2 | 1.80 | 2.32E-04 | 2.94E-02 |
| FMOD | 1.79 | 1.49E-04 | 2.17E-02 |
| EMILIN3 | -1.78 | 1.90E-04 | 2.52E-02 |
| CLEC2B | 1.78 | 2.51E-04 | 3.12E-02 |
| SV2B | -1.77 | 1.25E-04 | 1.87E-02 |
| NABP1 | 1.74 | 1.26E-04 | 1.87E-02 |
| CEBPD | 1.70 | 7.08E-05 | 1.23E-02 |
| CD14 | 1.68 | 8.40E-05 | 1.39E-02 |
| SLC11A1 | 1.67 | 1.83E-04 | 2.51E-02 |
| SIGLEC1 | 1.66 | 3.44E-04 | 4.03E-02 |
| CEBPB | 1.60 | 3.60E-04 | 4.18E-02 |
| SEZ6 | -1.59 | 1.93E-04 | 2.53E-02 |
| NTNG1 | 1.59 | 3.30E-04 | 3.91E-02 |
| PAPPA2 | 1.56 | 4.00E-04 | 4.56E-02 |
| VSTM2A | -1.53 | 1.85E-04 | 2.51E-02 |
| GLRX | 1.53 | 2.88E-04 | 3.51E-02 |
| PLTP | 1.51 | 2.53E-04 | 3.12E-02 |
| DUSP1 | 1.50 | 2.54E-04 | 3.12E-02 |
| SYNPO | 1.46 | 4.42E-04 | 4.91E-02 |
| VSIG4 | 1.46 | 3.70E-04 | 4.27E-02 |
| SLC1A2 | -1.46 | 4.51E-04 | 4.95E-02 |
| SOD2 | 1.43 | 3.07E-04 | 3.69E-02 |

**Supplementary Table 4**. Single cell sequencing of three matched pairs of HFC and LFC samples from IDH-WT glioblastoma patients.

| **Sample** | **Cells Before** | **Cells After** |
| --- | --- | --- |
| HFC 1 | 1277 | 967 |
| LFC 1 | 6671 | 4277 |
| HFC 2 | 2999 | 2529 |
| LFC 2 | 666 | 549 |
| HFC 3 | 4619 | 3170 |
| LFC 3 | 3914 | 2239 |
| **Total** |  | **13,731** |

Cells before and after QC/filtering. Filters chosen: 500 < nFeature_RNA < 10,000 & percent.mt < 0.2.

**Supplementary Table 5.** Demographics and clinical summary of patient cohorts used for survival data. *P* values determined by two-tailed Student’s t-test.

|  | **HFC-** | **HFC+** | ***P*-value** |
| --- | --- | --- | --- |
| No. of patients | 41 | 25 | - |
| Mean age (+/- SEM) | 60.6 (1.74) | 60.1 (2.52) | 0.82 |
| Gender (% male) | 53.7 | 60.0 | 0.61 |
| Tumor volume, cm^3^ (+/- SEM) | 21.4 (4.47) | 30.3 (4.52) | 0.19 |
| Extent of resection (%, +/- SEM) | 97.9 (50) | 96.8 (86) | 0.25 |
| IDH (% WT) | 41 (100) | 25 (100) | 1 |
| MGMT (% methylation) | 58.5 | 72.0 | 0.27 |
