# Supplementary material for: Glioblastoma remodelling of human neural circuits decreases survival
Source: Nature. 2023 May 3;617(7961):599–607. doi: 10.1038/s41586-023-06036-1 (PMC10191851; doi:10.1038/s41586-023-06036-1)
Supplement: Supplementary file 2 — Reporting Summary [file 41586_2023_6036_MOESM2_ESM.pdf]

nature portfolio

Corresponding author(s): Shawn Hervey-Jumper

Last updated by author(s): Jan 27, 2023

Reporting Summary

Nature Portfolio wishes to improve the reproducibility of the work that we publish. This form provides structure for consistency and transparency in reporting. For further information on Nature Portfolio policies, see our Editorial Policies and the Editorial Policy Checklist.

Statistics

For all statistical analyses, confirm that the following items are present in the figure legend, table legend, main text, or Methods section.

n/a	Confirmed	
☐	☒	The exact sample size (*n*) for each experimental group/condition, given as a discrete number and unit of measurement
☐	☒	A statement on whether measurements were taken from distinct samples or whether the same sample was measured repeatedly
☐	☒	The statistical test(s) used AND whether they are one- or two-sided<br>*Only common tests should be described solely by name; describe more complex techniques in the Methods section.*
☐	☒	A description of all covariates tested
☐	☒	A description of any assumptions or corrections, such as tests of normality and adjustment for multiple comparisons
☐	☒	A full description of the statistical parameters including central tendency (e.g. means) or other basic estimates (e.g. regression coefficient) AND variation (e.g. standard deviation) or associated estimates of uncertainty (e.g. confidence intervals)
☐	☒	For null hypothesis testing, the test statistic (e.g. *F*, *t*, *r*) with confidence intervals, effect sizes, degrees of freedom and *P* value noted<br>*Give P values as exact values whenever suitable.*
☒	☐	For Bayesian analysis, information on the choice of priors and Markov chain Monte Carlo settings
☒	☐	For hierarchical and complex designs, identification of the appropriate level for tests and full reporting of outcomes
☒	☐	Estimates of effect sizes (e.g. Cohen's *d*, Pearson's *r*), indicating how they were calculated

Our web collection on statistics for biologists contains articles on many of the points above.

Software and code

Policy information about availability of computer code

Data collection	Magnetoencephalography (MEG) recordings were analyzed using NUTMEG software suite version 4. For Bulk RNA-seq analysis, reads were trimmed with Trimmomatic and were mapped to the human reference genome GRCh38 using HISAT2 (v 2.1.0) and exon level count data were extracted from the mapped HISAT2 output using featureCounts. Differential gene expression analysis and log2 fold change calculation was performed using DESeq2. Confocal images were acquired using Nikon C2 confocal microscope and analyzed using Imaris Software (Imaris 9.2.1, Bitplane). Quantification of tumor cell burden was performed using a Zeiss LSM800 scanning confocal microscope and Zen 2011 imaging software (Carl Zeiss Inc.). Pre-operative and post-operative tumor volumes were quantified using BrainLab Smartbrush software (v 2.6) (Brainlab, Munich, Germany). Linear mixed effects modeling was used to perform statistical comparisons with repeated measures via the nlme package in R version 3.1-161 (https://cran.r-project.org/web/packages/nlme/citation.html). Multielectrode array (MEA) was collected using Axion integarted studio (AxIS) version 3.5.2 software.
Data analysis	Statistical analyses were conducted using Prism v8.0 (GraphPad software). Seurat R v3.0.1 was used for QC, analysis, and exploration of single-cell RNA-seq data. Confocal image analyses was done using Imaris 9.2.1 and Fiji ImageJ v2.0. Axion integarted studio (AxIS) version 3.5.2 software, Neural Metric Tool v1.2.3 software (Axion Biosystems) and Statistics Compiler function in AxIS was used for MEA analysis. Recursive partitioning analyses for survival data was performed using partDSA algorithm version 0.9.14.

For manuscripts utilizing custom algorithms or software that are central to the research but not yet described in published literature, software must be made available to editors and reviewers. We strongly encourage code deposition in a community repository (e.g. GitHub). See the Nature Portfolio guidelines for submitting code & software for further information.

Data

Policy information about availability of data

All manuscripts must include a data availability statement. This statement should provide the following information, where applicable:

- Accession codes, unique identifiers, or web links for publicly available datasets
- A description of any restrictions on data availability
- For clinical datasets or third party data, please ensure that the statement adheres to our policy

Bulk and single-cell RNA sequencing data of primary patient-derived samples reported in this manuscript are deposited in the NCBI Gene Expression Omnibus under the accession GSE223065. The publicly available GRCh38 (hg38, https://www.ncbi.nlm.nih.gov/assembly/GCF_000001405.39/) was used in this study. This manuscript contains no custom code or mathematical algorithms. All unique materials such as patient-derived cell cultures are available and can be obtained by contacting the corresponding author and with a standard MTA with University of California, San Francisco. The remaining data are available within the Article, and from Supplementary Information.

Human research participants

Policy information about studies involving human research participants and Sex and Gender in Research.

Reporting on sex and gender	The human subject data used in this work are all from adult males/females diagnosed with high-grade glioblastoma. Our findings apply to both sexes. A detailed information of patient's sex and age used in this study is provided in Extended Tables 2 and 5. Sex of patients was determined based on self reporting. We included sex as one of the prognostic variables besides other factors, such as age at diagnosis, tumor location, chemotherapy and radiotherapy to identify clinically relevant survival risk groups in a multivariate setting.
Population characteristics	The human subject data used in this work were all from adult males/females diagnosed with high-grade glioblastoma. Patient sample used for each experiment and clinical information of the patients used for human survival analysis is available in Extended Data Tables 1, 2 and 5.
Recruitment	All study participants were individuals seeking care for presumed diffuse glioma at University of California San Francisco. Each participant in this study was recruited from a prospective registry of adults aged 18–85 with newly diagnosed frontal, temporal, and parietal high-grade glioma. Inclusion criteria was patients with suspected brain tumor on magnetic resonance imaging (MRI). Exclusion criteria was any vulnerable population: children (age < 18). All human electrocorticography data was obtained during lexical retrieval language tasks from adult awake patients undergoing intraoperative brain mapping for surgical resection. Subjects with tumors projecting to the cortical surface as determined by absence of FLAIR or T1 post gadolinium enhancement were selected for analysis. All human magnetoencephalography recordings were obtained during resting state from adult patients aged 18–85 with newly diagnosed frontal, temporal, and parietal gliomas. Patients were recruited by a brain tumor center clinical research coordinator who was not involved in clinical patient care in order to limit the potential for enrollment bias.
Ethics oversight	This study complied with all relevant ethical regulations and was approved by the University of California, San Francisco (UCSF) institutional review board for human research (UCSF CHR 17-23215).

Note that full information on the approval of the study protocol must also be provided in the manuscript.

Field-specific reporting

Please select the one below that is the best fit for your research. If you are not sure, read the appropriate sections before making your selection.

☒ Life sciences ☐ Behavioural & social sciences ☐ Ecological, evolutionary & environmental sciences

For a reference copy of the document with all sections, see nature.com/documents/nr-reporting-summary-flat.pdf

Life sciences study design

All studies must disclose on these points even when the disclosure is negative.

Sample size	Patients presented for resection of glioma who gave consent for tumor sampling for research were included in the study, which was approved by the institutional review board (17-23215). No sample size calculation was performed, and all samples from patients meeting inclusion and exclusion criteria were included. Sample selection criteria are detailed in the Methods section.
Data exclusions	No data were excluded from the analyses.
Replication	All experiments were performed at least in triplicates and measurements were reproducible with biological replicates performed on separate cohort of patient samples/animals/cells.
Randomization	Primary Patient-derived tumor tissue biopsies and cells cultured from the tumor tissues were allocated based on imaging annotation for high

Randomization	functional connectivity (HFC) or low functional connectivity (LFC) experimental groups based on the mean imaginary coherence (IC) between the index MEG voxel, and the rest of the brain, referenced to its contralesional pair. All animals xenografted with individual cell lines used for immuno-electron microscopy and survival experiments were analyzed in the same way- no randomization was necessary. For pharmacological study, mice xenografted with HFC cells were randomized and intraperitoneally treated with gabapentin or corresponding vehicle.
Blinding	Investigators were blinded to the study groups being analyzed.

Reporting for specific materials, systems and methods

We require information from authors about some types of materials, experimental systems and methods used in many studies. Here, indicate whether each material, system or method listed is relevant to your study. If you are not sure if a list item applies to your research, read the appropriate section before selecting a response.

Materials & experimental systems

n/a	Involved in the study	
☐	☒	Antibodies
☐	☒	Eukaryotic cell lines
☒	☐	Palaeontology and archaeology
☐	☒	Animals and other organisms
☐	☒	Clinical data
☒	☐	Dual use research of concern

Methods

n/a	Involved in the study	
☒	☐	ChIP-seq
☒	☐	Flow cytometry
☐	☒	MRI-based neuroimaging

Antibodies

Antibodies used

Primary antibodies used in immunohistochemistry: chicken anti-neurofilament-H (1:1000; #NFH Aves Labs; Lot # NFH877982), chicken anti-neurofilament-M (1:1000; #NFM Aves Labs; NFM4907982), mouse anti-neurofilament antibody (1:1000; #NB300-134 Novus Biologicals; Lot # 021 521), rabbit anti-homer-1 (1:500; #PA5-21487 Pierce; Lot#TK2679033A) mouse anti-nestin (1:500; #Ab22035 Abcam; Lot#GR3276045-7), rabbit anti-synapsin 1 (1:1000; #AB1543 EMD Millipore; Lot#3051501), mouse anti-PSD95 clone K28/43 (1:100; #75-028 Neuromab; Lot# 455.7JD.22G), mouse anti-TSP-1 (1:20; #MA5-13398 Invitrogen), rabbit anti-TSP-1 (1:50, #Ab85762 Abcam; GR3279809-1) rabbit anti-MET (1:100; #Ab51067 Abcam; Lot#GR261314-23), rabbit anti-Ki67 (1:100; #Ab15580 Abcam; Lot#GR3293864-1), mouse anti-human nuclei clone 235-1 (HNA, 1:100, Millipore), chicken anti-MAP2 (1:500; #Ab15452 Abcam).

For secondary antibodies: Alexa 488 goat anti-chicken IgG (#A11039; Lot#1937504), Alexa 488 goat anti-rabbit IgG (#A11034; Lot#1971418), Alexa 568 goat anti-rabbit IgG (#A11036; Lot#1924788), Alexa 568 goat anti-mouse IgG (#A11004; Lot#1906485), Alexa 647 goat anti-rabbit IgG (#A21245; Lot#2051068) all used at 1:500 (Invitrogen).

Primary antibody used in immuno-electron microscopy: goat anti-RFP (1: 300; #ABIN6254205 Antibodies-online Inc.; Lot#0040180316) and secondary antibody (1:10; #15796 TED Pella; Lot#008330).

Validation

All the antibodies used in the study were bought from commercial vendors and were validated by the manufacturers, and/or in other studies:
Neurofilament (mouse, Novus Biological, NB300-134): Sikora J et al. X-linked Christianson syndrome: heterozygous female Slc9a6 knockout mice develop mosaic neuropathological changes and related behavioral abnormalities. Dis Model Mech. 2015. Validated in IHC by provider.

Homer (Pierce, PA5-21487): Gresa-Arribas N et al. Human neurexin-3α antibodies associate with encephalitis and alter synapse development. Neurology. 2016 Jun 14;86(24):2235-42. Validated in ICC/IF by provider.

Synapsin-1 (EMD Millipore, AB1543): Lin et al. Identification of diverse astrocyte populations and their malignant analogs. Nat Neurosci. 2017 Mar;20(3):396-405. Validated in ICC/IF by provider.

PSD95 (Neuromab, 75-028): Lin et al. Identification of diverse astrocyte populations and their malignant analogs. Nat Neurosci. 2017 Mar;20(3):396-405. Validated in ICC/IF by provider.

TSP-1 (Invitrogen, MA5-13398): Delaunay K et al. Meteorin Is a Novel Therapeutic Target for Wet Age-Related Macular Degeneration. J Clin Med. 2021 Jul 2;10(13):2973. Validated in IHC and IF by provider.

TSP-1 (Abcam, Ab85762): Zhang Y et al. Role of Elevated Thrombospondin-1 in Kainic Acid-Induced Status Epilepticus. Neurosci Bull 36:263-276 (2020). Validated in IHC-P, and ICC/IF by provider.

MET (Abcam, Ab51067): Hu H et al. Mutational Landscape of Secondary Glioblastoma Guides MET-Targeted Trial in Brain Tumor. Cell 175:1665-1678.e18 (2018). Validated in IHC-P by provider.

MAP2 (Abcam, Ab15452): Zabolocki M et al. BrainPhys neuronal medium optimized for imaging and optogenetics in vitro. Nat Commun 11:5550 (2020). Validated in ICC by provider.

RFP (Antibodies-online Inc., ABIN6254205): Semerci F et al. Lunatic fringe-mediated Notch signaling regulates adult hippocampal neural stem cell maintenance. Elife. 2017 Jul 12;6: e24660. Validated in IF and IHC-P by provider.

The chicken anti-neurofilament (M+H), mouse anti-nestin, rabbit anti-Ki67, and mouse anti-human nuclei used for immunohisto-chemical and immunocytochemistry analysis were all according to PMID: 31534222.

Eukaryotic cell lines

Policy information about cell lines and Sex and Gender in Research

Cell line source(s)	The eukaryotic cell lines SF#29, 31, 32, 33, 34, 35, 36, 44, 45,49, 51, 53, 54, 56, 57, 59, 60, 62 and 63 derived from primary patient-derived high-grade gliomas were generated in the Shawn Hervey-Jumper lab from site-directed biopsies (HFC/LFC), and referenced in the Supplementary Table 1 of the manuscript.
Authentication	Short Tandem Repeat (STR) fingerprinting is performed every 3 months on all cell cultures to ensure authenticity.
Mycoplasma contamination	All cell cultures are routinely tested for mycoplasma contamination and all cultures were tested negative.
Commonly misidentified lines (See ICLAC register)	No commonly misidentified lines were used.

Animals and other research organisms

Policy information about studies involving animals; ARRIVE guidelines recommended for reporting animal research, and Sex and Gender in Research

Laboratory animals	NOD-SCID-IL2R gamma chain-deficient (NSG) and female athymic mice were used between 4-12 weeks of age. Mouse housing conditions have been described in the manuscript.
Wild animals	No wild animals were used.
Reporting on sex	Both male and female NOD-SCID-IL2R gamma chain-deficient (NSG) and female athymic mice were used between 4-12 weeks of age.
Field-collected samples	No field-collected samples were used.
Ethics oversight	Study was approved by UCSF Institutional Animal Care and Use Committee (AN192389-01G).

Note that full information on the approval of the study protocol must also be provided in the manuscript.

Clinical data

Policy information about clinical studies

All manuscripts should comply with the ICMJE guidelines for publication of clinical research and a completed CONSORT checklist must be included with all submissions.

Clinical trial registration	*Provide the trial registration number from ClinicalTrials.gov or an equivalent agency.*
Study protocol	Each participant in the study was recruited from a prospective registry of adults aged 18–85 with newly diagnosed frontal, temporal, and parietal IDH-wild type high-grade gliomas with detailed language assessments and baseline MEG recordings. Inclusionary criteria included the following: native English-speaking, between the ages of 18- 85, and no prior history of psychiatric illness, neurologic illness or drug or alcohol abuse. All human electrocorticography data were obtained during lexical retrieval language tasks from 14 adult awake patients undergoing intraoperative brain mapping for surgical resection. Tumors from 8 patients were used for RNA-sequencing experiments. Site-directed tumor biopsies from 19 patients were used for immunofluorescence/immunohistochemistry analysis and 24 patients were used for immunocytochemistry and cell-based functional assays. Tumors from 8 patients were used for mouse xenograft experiments. All participants provided written informed consent to participate in this study, which was approved by the University of California, San Francisco (UCSF) institutional review board (IRB) for human research (UCSF CC-171027, CHR 17-23215) and performed in accordance with the Declaration of Helsinki.
Data collection	Language assessments including picture naming, text reading, auditory naming, syntax, and 4 syllable repetition are collected by a rained clinical research coordinator at the time of initial diagnosis as well as during the intra operative setting during standard of care clinical protocol.
Outcomes	Primary outcomes include auditory and picture naming task performance, scored according to Wilson et al (Wilson, S. M., Eriksson, D. K., Schneck, S. M., & Lucanie, J. M. (2018). A quick aphasia battery for efficient, reliable, and multidimensional assessment of language function. PLoS ONE, 13(2), e0192773. http://doi.org/10.1371/journal.pone.0192773)

Magnetic resonance imaging

Experimental design

Design type	Resting state

Design specifications

An artifact-free, 1-minute epoch was analyzed using the NUTMEG software suite (UCSF Biomagnetic Imaging Laboratory) to reconstruct whole-brain oscillatory activity from MEG sensors so as to construct functional connectivity (imaginary coherence) metrics.

Behavioral performance measures

MRI/MEG was aquired as a means of measuring imaginary coherence functional connectivity. There were no behavioral outcomes assessed in the MRI portion of this study.

Acquisition

Imaging type(s)

Structural MRI. Imaging Acquisition and MEG Data Analysis High-resolution MRI was performed using a 3-T unit to provide anatomical detail.

Field strength

3T

Sequence & imaging parameters

The protocol included the following sequences: 1) a T1-weighted, 3D, spoiled gradient–recalled acquisition in steady-state sequence, with TR 6–9 msec, TE 2–3 msec, and flip angle 12°–15°; and 2) a T2-weighted, 3D, fast spin echo sequence, with TR 2000–3800 msec and TE 87–159 msec. Both sequences had a slice thickness between 1 and 1.5 mm, an acquisition matrix from 256 × 256 to 288 × 288, contained between 114 and 428 slices. Patients were lying awake with their eyes closed in a magnetically shielded room while a 275-channel whole-head CTF Omega 2000 system (CTF Systems, Inc.) captured their continuous resting-state MEG using a sampling rate of 1200 Hz. The locations of the MEG coils were triangulated at the beginning and end of the recording run and later coregistered to a structural MR image to generate the head shape.

Area of acquisition

Whole brain

Diffusion MRI ☐ Used ☒ Not used

Preprocessing

Preprocessing software

Data were analyzed using the NUTMEG software suite.

Normalization

Normalization was visualized using SPM and checked by eye for proper normalization.

Normalization template

MNI

Noise and artifact removal

The time series within each voxel was then bandpass filtered for the alpha band (1–20 Hz) and reconstructed in source space using a minimum-variance adaptive spatial filtering technique.

Volume censoring

60 second noise free periods were chosen for analysis.

Statistical modeling & inference

Model type and settings

Functional connectivity estimates were calculated using IC, a technique known to reduce overestimation biases in MEG data generated from common references, cross-talk, and volume conduction.

Effect(s) tested

The alpha band was selected because it was the most consistently identified peak in the power spectra from this sampling window in our patient series. The imaginary coherence technique was used to estimate functional connectivity because it has been previously been shown to reduce overestimation biases in MEG data.

Specify type of analysis: ☒ Whole brain ☐ ROI-based ☐ Both

Statistic type for inference
(See Eklund et al. 2016)

A univariate regression model was generated to analyze each of the independent variables to determine significance. A significance level of $p < .05$ was used. Statistical testing was conducted

Correction

FDR

Models & analysis

n/a	Involved in the study
☐	☒ Functional and/or effective connectivity
☒	☐ Graph analysis
☐	☒ Multivariate modeling or predictive analysis

Functional and/or effective connectivity

Imaginary Coherence

Multivariate modeling and predictive analysis

To identify clinically relevant survival risk groups in a multivariate setting, we employed recursive partitioning analyses for survival data via the partDSA algorithm. Survival trees use recursive partitioning to divide patients into different risk groups. The Brier score was the chosen loss function for splitting and pruning. Such methods are non-parametric and, therefore, do not require the proportional hazards assumption. All known prognostic variables were included in the trees, including age at diagnosis, sex, tumor location, chemotherapy, radiotherapy, the presence of functional connectivity within the tumor, pre- and post-

operative tumor volume, and EOR. The tree that minimized the five-fold cross-validated error as well as the most parsimonious tree within one standard error of the overall minimum error were selected for review. Leaves of the resulting trees defined the final risk groups from which the corresponding Kaplan-Meier curves were generated. Median OS times and hazard ratios were generated and compared between risk groups using the Kaplan-Meier method and Cox proportional hazards model, respectively. The proportional hazards assumption was verified.
